# Predictors of HVTN 503 MRK-AD5 HIV-1 gag/pol/nef Vaccine Induced Immune Responses

**DOI:** 10.1371/journal.pone.0103446

**Published:** 2014-08-04

**Authors:** Kathryn L. Hopkins, Fatima Laher, Kennedy Otwombe, Gavin Churchyard, Linda-Gail Bekker, Stephen DeRosa, Maphoshane Nchabeleng, Koleka Mlisana, James Kublin, Glenda Gray

**Affiliations:** 1 Perinatal HIV Research Unit, Faculty of Health Sciences, University of the Witwatersrand, Johannesburg, South Africa; 2 Aurum Institute for Health Research, Faculty of Health Sciences, School of Public Health, University of the Witwatersrand, Johannesburg, South Africa; 3 Desmond Tutu HIV Centre, University of Cape Town, Cape Town, South Africa; 4 HIV Vaccine Trials Network, Fred Hutchinson Cancer Research Center, Seattle, Washington, United States of America; 5 Medunsa HIV Clinical Research Unit, University of Limpopo, Pretoria, South Africa; 6 Centre for AIDS Programme for Research in South Africa & Department of Medical Microbiology, University of KwaZulu-Natal, Durban, South Africa; Mayo Clinic, United States of America

## Abstract

**Background:**

Phambili, the Merck (MRK)-Adenovirus Type 5 (Ad5) HIV-1 gag/pol/nef subtype B vaccine study, conducted in South Africa, suspended enrollment and vaccination when companion study, Step, was found non-efficacious. Although the vaccine did not prevent HIV-1 infection or lower viral-load setpoint, immune responses recognized clades B and C HIV-1 subtypes. We investigated predictors of the vaccine-induced antigen-specific immune responses.

**Methods:**

Vaccine-induced immunogenicity was ascertained by interferon-γ ELISpot assays on the first 186 enrolled participants receiving two vaccinations. Analyses, stratified by study arm/sex, were performed on baseline demographics [sex, age, Body Mass Index (BMI), site, Adenovirus Type-5 (Ad5) titer, Herpes Simplex Virus Type-2 (HSV2) status, heavy drinking]. Multivariate logistic regression determined predictors.

**Results:**

Of the 186 participants, 53.7% (n = 100) were female, median BMI was 22.5 [IQR: 20.4–27.0], 85.5% (n = 159) were Ad5 seropositive, and 18.8% (n = 35) drank heavily. All vaccine recipients responded to both clade B (n = 87; 47%) and/or C (n = 74; 40%), p = 0.17. In multivariate analysis, female sex [Adjusted Odds Ratio (AOR): 6.478; p = 0.0159], overweight/obese BMI (AOR: 0.186; p = 0.0452), and heavy drinking (AOR: 0.270; p = 0.048) significantly predicted immune response to clade C for any antigens. A marginally significant predictor of clade C-pol antigen was female sex (AOR: 3.182; p = 0.0500).

**Conclusions:**

Sex, BMI, and heavy drinking affected vaccine-induced HIV-1 specific immune responses to clade C antigens. The role of female sex and overweight/obese BMI boosting and suppressing vaccine-induced HIV-1 specific immune responses, respectively, requires elucidation, including any effect on HIV vaccine efficacy, especially in the era of colliding epidemics (HIV and obesity).

## Introduction

Phambili (HVTN 503) was a Phase IIb HIV vaccine efficacy study conducted in South Africa. It investigated the Merck (MRK)-Adenovirus type-5 (Ad5) HIV-1 clade B trivalent HIV vaccine, which contains recombinant Ad5 (rAd5) vectors expressing clade B gag, pol, and nef epitopes [1]–[3]. The trial suspended enrollment and vaccination in September 2007 when the interim efficacy analysis from its companion study, Step (HVTN 502/MRK 023), conducted in North America, South America, the Caribbean, and Australia, crossed pre-specified futility boundaries for efficacy while still in its vaccination period [1],[3]. Although the vaccine neither prevented HIV-1 infection nor lowered viral load setpoint, immune responses recognized clades B and C HIV-1 subtypes [3].

Vaccine-induced immune responses to HIV-1 may not necessarily equate to protection or risk. It is uncertain which immune responses are required for protection against HIV infection or controlling viremia [4]. Most vaccines combating viruses stimulate production of antigen-specific memory B- and/or cytotoxic T lymphocytes (CTL) which can rapidly multiply upon exposure to targeted pathogens, controlling infection. Ad5 is the best-studied serotype of adenoviral vaccine vectors [5]. Replication-defective Ad5 viruses are potential HIV vaccine vectors based on their ability to stimulate anti-HIV CTL responses associated with killing HIV-infected cells following initial infection [5]. Phase 1 clinical studies found Ad5 vector-based vaccines to be among the most immunogenic of cell-mediated immune vaccines [6], [7]. Gag, pol, and nef epitopes are known to elicit high CTL responses, helping control HIV; gag proteins create structural HIV membrane components, and pol and nef proteins support viral replication [8].

The MRK-Ad5 HIV-1 gag/pol/nef vaccine, consisting of a 1∶1∶1 mixture of these three vectors, expresses the gag gene from the HIV-1 strain CAM-1, the pol gene from HIV-1 strain IIIB, and the nef gene from HIV-1 strain JR-FL. These genes are representative of the respective consensus clade B sequences. Each vector expresses one of the three genes inserted into the adenoviral E1 region. This trivalent vaccine presently consists of mixtures of the three individual vectors. The MRK-Ad5 HIV-1 gag/pol/nef trivalent vaccine elicited gag-specific immune responses comparable to those elicited by the Ad5 and MRK-Ad5 HIV-1 gag monovalent vaccines, but the responses elicited by the trivalent vaccine were broader since responses were also seen to both pol and nef peptides. Broader responses should make immunological escape less likely to occur. The MRK-Ad5 vaccine did stimulate CTL responses in a large proportion of vaccine-recipients, but failed to prevent HIV acquisition and control HIV levels in participants who became infected during the trial [3]. It remains unknown if the MRK-Ad5 vaccine efficacy failure was due to the vaccine regimen or whether CTL properties simply are not useful correlates of immune protection [4].

Another challenge in developing an effective HIV vaccine is the paucity of data to elucidate the innate predictors affecting immune responses and to understand how this could affect vaccine efficacy [9]. Previous studies of viral vector or recombinant subunit vaccines provide evidence of socio-demographic predictors of immune response, such as age and obesity [10]–[14]. Influenza vaccinations with inactivated virus are less effective, eliciting poor immune responses, in both young children and the elderly, due to either undeveloped or less-proficient, aged immune systems, respectively [10]–[12]. One study showed only 17% of 153 participants aged 65–98 years receiving a trivalent influenza vaccine generated increased antibody titers to all three vaccine components, and 46% of those immunized failed to respond to all three of the vaccine elements [10]. Obesity, as measured by a higher weight-height index, and older age were predictors of poor immunogenic response to the Hepatitis B plasma vaccine [13]. A higher dosage series of the Hepatitis B vaccine induced greater protective levels of antibodies in 94–100% of healthy infants, children, and adolescents; yet immune responses for the same three-dose vaccination series were lower for older persons (adults aged 40–60 years, >90% protection; adults aged 60+ years, 65–75% protection) [14].

Phambili participant risk behavior at baseline or during the study served as unlikely explanations for the increased rate of HIV-1 infections seen in vaccine recipients [15]. It is not yet known if there are innate, influential factors affecting the Phambili vaccine-induced immune response. We investigate predictors associated with the MRK-Ad5 vaccine-specific T-cell immune response for HIV-1 clade B and clade C amongst South African participants in an attempt to further improve upon future HIV vaccine candidates.

## Methods

### Study Design of Phambili and Participants

The methods of Phambili have been previously described [3]. In summary, Phambili was a multicenter, double-blind, randomized, placebo-controlled clinical trial conducted between 24 January and 19 September, 2007. The interim efficacy analysis report found the study vaccine elicited an immune response recognizing two subtypes of HIV-1 (clade B and C) but was not deemed efficacious [3]. The clinical trial was approved by the relevant regulatory bodies in South Africa and the United States. Participants provided written informed consent in English or their local language [3]. The trial was registered in clinicaltrials.gov (NCT00413725) and the South Africa National Health Research Database (DOH-27-0207-1539) [3].

Phambili was conducted at five sites: Soweto, Cape Town, Klerksdorp, eThekwini, and Medunsa. Eligible individuals were consenting, healthy, HIV-1-uninfected men and women aged 18–35 years, sexually active within six months of enrollment. Women could not be pregnant or breastfeeding, and agreed to use two methods of contraception [3]. Heavy drinking in the previous six months, an HIV-1 risk behavior, was assessed at screening [3].

### Participant Procedures

Of 1428 individuals screened, 801 were randomized to receive either vaccine or placebo (400 vaccine recipients, 401 placebo-controls) [3]. Participants were randomized 1∶1 between vaccine and placebo, using blocked randomization stratified across sites and sex [3].

Vaccines were injected intramuscularly at 0, 1, 6 months, with 1.5×10^10^ adenovirus genomes per mL of study product or 1.0 mL solution of vaccine diluent without Ad5 vector as placebo [3].

Participant serum samples were collected at enrollment for neutralizing Ad5 (NAd5) antibody titers [3] and herpes simplex virus type-2 (HSV-2) serology [3]. The HSV-2 antibody is a marker of previous exposure to a common sexually transmitted infection that has been associated with HIV-acquisition, especially in urban African populations [16]. Clinical assessments were performed and HIV-1 prevention interventions were provided throughout the trial. HIV-1 testing was conducted at first vaccination, week 12, week 30, at unmasking, and every six months thereafter during the follow-up period with an algorithm determining true infection from vaccine-induced seropositivity [3], [17].

### Immunological Tests

Since interferon-γ (IFN-γ) ELISpot was the primary immunogenicity assay used for early phase vaccine testing, it remained as such for this study. At the Fred Hutchinson Research Center HVTN laboratory, vaccine immunogenicity was assessed on a subset of participants using previously cryopreserved peripheral-blood mononuclear cells (PBMC) obtained by venipuncture at week 8, four weeks post second vaccination [3]. A validated IFN-γ ELISpot assay assessed the ex-vivo T-cell responses using PBMC stimulated overnight with two panels of peptide pools from HIV-1 clades B and C, using standard protocols [1]. There were peptide pools for two clades because the vaccine encoded clade B antigens while the study was conducted in a clade C region; there was also interest in the ability of the vaccine to elicit cross-protective clade C responses. The synthetic clade B peptide pools contained vaccine-matched proteins – one gag, one nef, and two pol pools. Since vaccine-matched peptides were not an option to evaluate clade C responses, the clade C peptide pools contained potential T-cell epitopes (PTE) of common clade C peptides from Southern African isolates – two gag, one nef, and three pol pools [3].

The assay was conducted for the first 186 participants enrolled (93 vaccine recipients, 93 placebo-controls) who were HIV-1 antibody negative at the week 12 visit, received the first vaccination and then second injection within the day 28 visit window (days 19–42), and whose thawed PBMC had ≥66% viability [3]. Specimens included in the statistical analysis met the following criteria: mean of the medium-only wells ≤ six background-subtracted spot forming cells (SFCs)/200,000 PBMC; mean for the three positive control wells ≥400 SFCs/200,000 PBMC; mean for the negative control wells ≤20 SFCs/200,000 PBMC; and results existed for at least four of the six negative control wells [3]. Lab personnel were blinded to the treatment arm assignment.

### Statistical Methodology

Statistical analyses were performed with SAS (version 9.3, SAS Institute, Cary, NC). The ELISpot assay analysis methodology was previously described [3].

BMI (measured as: weight (in kilograms) / [height (in meters)]^2^) was categorized as the following: underweight <18.5; normal 18.5–24.9; overweight 25.0–29.9; and obese >29.9. NAd5 titer was defined as detectable (≥18 units) and undetectable (<18 units). Heavy drinking was defined per protocol as having more than five drinks per day on at least ten days within the past six months.

Baseline demographical information (study site, age, BMI, Ad5 titer, HSV-2 status, and heavy drinking) for the 186 immunogenicity participants was presented as frequencies for categorical variables and descriptively for continuous variables, stratified by study arm and sex. Chi-squared analyses compared categorical variables. BMI was evaluated by sex and across sites using the Kruskall-Wallis non-parametric test.

Univariate and multivariate predictors of clades B and C immune responses for both any and clade-specific antigens were determined for the 93 vaccine recipients. The multivariate model was adjusted for site and age-group. Backward-selection technique was performed for multivariate variable selection. Hosmer-Lemeshow Goodness of fit statistics established model diagnostics. Each two-tailed analysis was determined at the 5% level of significance. There were no missing data.

## Results

Our paper describes a subset of enrolled participants, 93 per study arm, assessed for immunogenicity by ELISpot analysis.

### Baseline Demography of Immunogenicity Participants

Table 1 describes all baseline demographics. Of the 186 immunogenicity participants, 100 (53.7%) were female [51 (51.0% of 100) were vaccine recipients], and 86 (46.3%) were male [42 (48.8% of 86) were vaccine recipients]. Due to staggered site initiation, the immunogenicity cohort resided in only three catchment areas – Soweto (n = 100; 53.8%), Cape Town (n = 45; 24.2%), and Klerksdorp (n = 41; 22.0%). Median age was 23 years [Interquartile Range (IQR): 21–27]. Most participants were ≤25 years old (n = 121; 65.1%). Median BMI was 22.5 [IQR: 20.4–27.0]. Females had a higher median BMI than males (25.3 vs. 20.9; p<0.0001). Most participants had BMI <25.0 (n = 122; 65.6%). Cape Town had the highest BMI (n = 45; median 28.0 [IQR: 23.3–32.4]), followed by Soweto (n = 100; median 22.0 [IQR: 20.2–25.3]), then Klerksdorp (n = 41; median 20.7 [IQR: 19.1–22.1]). While not shown in Table 1, all mean BMI comparisons among sites were significant at the 5% level.

**Table 1:** Baseline Demography of Immunogenicity Participants at Enrollment.

	Vaccine Arm (n = 93)		Placebo Arm (n = 93)		
	Males	Females	Males	Females	Total
	(n = 42, 45%)	(n = 51, 55%)	(n = 44, 47%)	(n = 49, 53%)	(N = 186)
**Demographics**					
**Study Site, n (%)**					
Soweto	22 (52.4)	29 (56.9)	20 (45.5)	29 (59.2)	100 (53.8)
Klerksdorp	12 (28.6)	7 (13.7)	16 (36.4)	6 (12.2)	41 (22.0)
Cape Town	8 (19.0)	15 (29.4)	8 (18.2)	14 (28.6)	45 (24.2)
**Median Age, yrs**	23	23	23	24	23
**(IQR)**	(21.0–27.0)	(20.0–27.0)	(21.0–27.0)	(21.0–28.0)	(21.0–27.0)
					
**Median BMI**	20.9	25.0	20.9	25.5	22.5
**(IQR)**	(18.8–22.3)	(21.6–29.7)	(19.7–23.2)	(22.2–30.4)	(20.4–27.0)
					
**Median BMI by Site (IQR)**					
Soweto	20.3 (18.3–21.0)	23.6 (21.3–26.7)	20.7 (19.8–22.8)	25.2 (22.5–28.6)	22.0 (20.2–25.3)
Klerksdorp	21.2 (19.2–22.2)	21.4 (17.0–27.4)	19.9 (19.3–21.0)	20.9 (18.1–25.5)	20.7 (19.1–22.1)
Cape Town	23.0 (22.3–24.7)	29.5 (26.6–31.6)	28.6 (22.9–31.2)	31.6 (25.1–42.0)	28.0 (23.3–32.4)
**Ad5 Titer, n (%)**					
<18	11 (26.2)	7 (13.7)	4 (9.1)	5 (10.2)	27 (14.5)
18–200	10 (23.8)	16 (31.4)	13 (29.5)	13 (26.5)	52 (28.0)
201–1000	13 (31.0)	20 (39.2)	21 (47.7)	20 (40.8)	74 (39.8)
>1000	8 (19.0)	8 (15.7)	6 (13.6)	11 (22.4)	33 (17.7)
**HSV-2 Status, n (%)**					
Positive	11 (26.2)	31 (60.8)	6 (13.6)	26 (53.1)	74 (39.8)
Negative	31 (73.8)	20 (39.2)	36 (81.8)	23 (46.9)	110 (59.1)
Atypical	0 (0.0)	0 (0.0)	2 (4.5)	0 (0)	2 (1.1)
**Heavy Drinking** a **, n (%)**					
Yes	15 (35.7)	3 (5.9)	12 (27.3)	5 (10.2)	35 (18.8)
No	27 (64.3)	48 (94.1)	32 (72.7)	44 (89.8)	151 (81.2)

Abbreviations: IQR, Interquartile Range; BMI, Body Mass Index; Ad5, Adenovirus Type-5; HSV-2, Herpes Simplex Virus Type-2.

a Heavy drinking was self-reported and defined as having more than five drinks per day on at least ten days within six months of the screening and enrollment period.

The majority of all trial participants was Ad5 antibody positive (n = 159; 85.5%). The proportion of Ad5 sero-negative males was higher in vaccine recipients as compared to placebo-recipients (11 vs. 4; p = 0.037). HSV-2 seropositivity at baseline was 39.8%. Females were more likely to be HSV-2 positive at baseline (57 vs. 17; p<0.0001). Heavy drinkers comprised 18.8% of the cohort: males were more likely to report heavy drinking than females (27 vs. 8; p<0.0001).

### ELISpot Results for any Clade B or C Antigen and gag/pol/nef-Specific Responses

As shown in Table 2, 87 (46.8%) of 186 immunogenicity participants responded to either clade B or clade C antigens (83 [89.2%] of 93 vaccine recipients). All vaccine recipients developed an IFN-γ-secreting T-cell response to either clade B (n = 83; 89.2%) and/or clade C peptides (n = 72; 77.4%; p = 0.17).

**Table 2:** Distribution of ELISpot Response per Clade for Any Antigen and Specific Antigens.

	Vaccine Arm (n = 93)		Placebo Arm (n = 93)		
	Males (n = 42, 45%)	Females (n = 51, 55%)	Males (n = 44, 47%)	Females (n = 49, 53%)	Total (N = 186)
**ELISpot Response**					
**Any ELISpot Response, n (%)**					
Non-Responder	6 (14.3)	4 (7.8)	43 (97.7)	46 (93.9)	99 (53.2)
Responder^a^	36 (85.7)	47 (92.2)	1 (2.3)	3 (6.1)	87 (46.8)
**Clade B ELISpot Response, n (%)**					
Non-responder	6 (14.3)	4 (7.8)	43 (97.7)	46 (93.9)	99 (53.2)
Responder^a^	36 (85.7)	47 (92.2)	1 (2.3)	3 (6.1)	87 (46.8)
**Clade C ELISpot Response, n (%)**					
Non-responder	14 (33.3)	7 (13.7)	44 (100.0)	47 (95.9)	112 (60.2)
Responder^a^	28 (66.7)	44 (86.3)	0 (0.0)	2 (4.1)	74 (39.8)
**Clade B-gag ELISpot Response, n (%)**					
Non-responder	10 (23.8)	9 (17.7)	44 (100.0)	46 (93.9)	109 (58.6)
Responder^a^	32 (76.2)	42 (82.4)	0 (0.0)	3 (6.1)	77 (41.4)
**Clade C-gag ELISpot Response, n (%)**					
Non-responder	20 (47.6)	25 (49.0)	44 (100.0)	47 (95.9)	136 (73.1)
Responder^a^	22 (52.4)	26 (51.0)	0 (0.0)	2 (4.1)	50 (26.9)
**Clade B-pol ELISpot Response, n (%)**					
Non-responder	16 (38.1)	11 (21.6)	43 (97.7)	47 (95.9)	117 (62.9)
Responder^a^	26 (61.9)	40 (78.4)	1 (2.3)	2 (4.1)	69 (37.1)
**Clade C-pol ELISpot Response, n (%)**					
Non-responder	15 (35.7)	13 (25.5)	44 (100.0)	48 (98.0)	120 (64.5)
Responder^a^	27 (64.3)	38 (74.5)	0 (0.0)	1 (2.0)	69 (35.5)
**Clade B-nef ELISpot Response, n (%)**					
Non-responder	14 (33.3)	14 (27.4)	44 (100.0)	46 (93.9)	118 (63.4)
Responder^a^	28 (66.7)	37 (72.6)	0 (0.0)	3 (6.1)	68 (36.6)
**Clade C-nef ELISpot Response, n (%)**					
Non-responder	32 (76.2)	41 (80.4)	44 (100.0)	48 (98.0)	165 (88.7)
Responder^a^	10 (23.8)	10 (19.6)	0 (0.0)	1 (2.0)	21 (11.3)

a Responders were defined as those with antigen-stimulated responses significantly greater than twice their background responses as assessed by a bootstrap test (one-tailed α 0.05) after adjusting for the multiple antigens within each clade; additionally, background-subtracted responses had to exceed ten SFC per 200 000 PBMC [3].

Table 2 also shows the distribution of ELISpot responses for gag/pol/nef-specific antigens. For vaccine recipients, there was a greater clade B response compared to clade C for antigens gag (74 vs. 48; p = 0.0001) and nef (65 vs. 20; p<0.0001). Clade B and C responses were similar for the pol-antigen (66 vs. 65; p = 0.87). Amongst female vaccine recipients, there was a greater B-gag compared to C-gag response (82.4% vs. 51.0%; p = 0.0008); and greater B-nef compared to C-nef response (72.6% vs. 19.6%; p<0.0001).

### Multivariate Analyses

In multivariate analysis (Table 3), a statistically significant predictor for a positive clade C immune response was female sex [Adjusted Odds Ratio (AOR): 6.478; CI: 1.419–29.570; p = 0.0159]. Having an overweight/obese BMI [AOR: 0.186; CI: 0.036–0.965; p = 0.0452] or being a heavy drinker [AOR: 0.270; CI: 0.073–0.990; p = 0.0483] significantly suppressed the same immune response.

**Table 3:** Predictors of Phambili Vaccine Immune Response for any Clade C Antigens.

	Clade C Univariate		Clade C Multivariate	
	OR (95% CI)	p value	OR (95% CI)	p value
**Sex**				
Male	1.0		1.0	
Female	3.14 (1.13–8.75)	0.02	6.48 (1.42–29.57)	0.01
**Study Site**				
Soweto	0.53 (0.19–1.46)	0.22	0.31 (0.09–1.07)	0.06
Others	1.0		1.0	
**Age Group**				
≤24 years	1.0		1.0	
>24 years	1.35 (0.49–3.75)	0.57	1.07 (0.34–3.44)	0.90
**BMI** a				
Normal/Underweight	1.0		1.0	
Overweight/Obese	1.03 (0.35–3.01)	0.96	0.19 (0.04–0.97)	0.04
**Ad5 Titer^b^**				
Positive	0.63 (0.16–2.44)	0.51	0.35 (0.08–1.60)	0.18
Negative	1.0		1.0	
**Heavy Drinking^c^**				
Yes	0.26 (0.09–0.79)	0.01	0.27 (0.07–0.99)	0.04
No	1.0		1.0	

Abbreviations: OR, Odds Ratio; CI, Confidence Interval; BMI, Body Mass Index; Ad5, Adenovirus Type-5.

a BMI was categorized as underweight (<18.5), normal (18.5–24.9), overweight (25.0–29.9), and obese (>29.9); ^b^Ad5 titer levels were defined as positive (>18 units) and negative (≤18 units); and ^c^Heavy drinking was self-reported and defined as having more than five drinks per day on at least ten days within six months of the screening and enrollment period.

All categorical references are valued as 1.0. Bold values represent statistically significant data.

Multivariate analysis showed only one significant antigen-specific immune response. Table 4 shows female sex is moderately significant in predicting an increased immune response to clade C-pol antigens (AOR: 3.182; CI: 1.000–10.120; p = 0.0500). Age, BMI, Ad5 titer, site, and heavy drinking did not predict a significant antigen-specific immune response.

**Table 4:** Predictors of Phambili Vaccine Immune Response for Clade-C-Specific Antigens.

	Gag Univariate		Gag Multivariate		Pol Univariate		Pol Multivariate		Nef Univariate		Nef Multivariate	
	OR (95%CI)	p value	OR (95% CI)	p value	OR (95% CI)	p value	OR (95% CI)	p value	OR (95% CI)	p value	OR (95% CI)	p value
**Gender**												
Male	1.0		1.0		1.0		1.0		1.0		1.0	
Female	0.95 (0.42–2.14)	0.89	1.14 (0.40–3.21)	0.81	1.62 (0.67–3.96)	0.29	3.18 (1.00–10.12)	0.05	0.78 (0.29–2.10)	0.62	0.79 (0.24–2.62)	0.70
**Study Site**												
Soweto	0.67 (0.29–1.52)	0.33	0.58 (0.24–1.45)	0.25	0.57 (0.23–1.43)	0.23	0.41 (0.15–1.11)	0.08	1.01 (0.37–2.73)	0.99	–	–
Others	1.0		1.0		1.0		1.0		1.0		–	–
**Age Group**												
≤24 years	1.0		1.0		1.0		–	–	1.0		1.0	
>24 years	1.30 (0.56–2.99)	0.55	1.24 (0.50–3.05)	0.64	1.50 (0.59–3.82)	0.39	–	–	1.39 (0.51–3.79)	0.52	1.45 (0.52–4.06	0.48
**BMI^a^**												
Normal/Underweight	1.0		1.0		1.0		1.0		1.0		1.0	
Overweight/Obese	0.67 (0.27–1.64)	0.38	0.45 (0.15–1.37)	0.16	0.81 (0.31–2.11)	0.66	0.37 (0.11–1.30)	0.12	0.77 (0.25–2.39)	0.65	0.74 (0.20–2.79)	0.66
**Ad5 Titer^b^**												
Positive	0.62 (0.22–1.77)	0.38	0.55 (0.18–1.66)	0.29	0.40 (0.11–1.51)	0.18	0.30 (0.07–1.20)	0.09	0.65 (0.20–2.11)	0.47	0.65 (0.19–2.16)	0.48
Negative	1.0		1.0		1.0		1.0		1.0		1.0	
**Heavy Drinking^c^**												
Yes	0.53 (0.18–1.51)	0.23	0.41 (0.13–1.34)	0.14	0.45 (0.16–1.31)	0.15	–	–	0.68 (0.18–2.64)	0.58	0.54 (0.12–2.33	0.40
No	1.0		1.0		1.0		–	–	1.0		1.0	

Abbreviations: OR, Odds Ratio; CI, Confidence Interval; BMI, Body Mass Index; Ad5, Adenovirus Type-5. ^a^BMI was categorized as underweight (<18.5), normal (18.5–24.9), overweight (25.0–29.9), and obese (>29.9); ^b^Ad5 titer levels were defined as positive (>18 units) and negative (≤18 units); and ^c^Heavy drinking was self-reported and defined as having more than five drinks per day on at least ten days within six months of the screening and enrollment period.

All categorical references are valued as 1.0, and missing data indicate that the demographic was not included in the multivariate regression model. Bold values represent statistically significant data.

## Discussion

This is the first investigation of predictors of immune response to the Phambili vaccine. Our study demonstrates certain demographic factors affect the immunologic response to clade C antigens, only. Female sex significantly boosts immune response to any clade C antigen. Heavy drinking and overweight/obese BMI significantly inhibit this immune response. Female sex is marginally significant in boosting immune response to the clade C-pol antigen. Baseline NAd5 titer levels were not a statistically significant predictor of vaccine immune response.

All 93 vaccine recipients showed immune responses to either clade B and/or clade C antigens, confirming the Phambili clade B vaccine had cross-clade reactivity. As previously reported, all clade C responders also had a clade B response, and gag-specific responses were highest for clade B, as pol-specific responses were for clade C [3]. Consistent with the regional clade prevalence, there were only significant predictors to a clade C immune response.

This study adds to existing evidence that cross-clade immune responses are possible. A study conducted in Brazil, Malawi, South Africa, Thailand, and the USA looked at the cross-reactivity of the anti-HIV-1 T-cell responses using IFN-γ ELISpot assays major viral clades [18]. It showed that T-cell immune responses exhibited a substantial degree of cross-reactivity. In South Africa, 95% of the population responded to clade B gag and/or nef proteins [18].

We did not look at magnitude of immune response (median SFCs per 10^6^ PBMC), just presence. However, to summarize previous reporting among responders: any response (678 clade B vs. 470 clade C), gag (181 vs. 168), pol (292 vs. 250), and nef (168 vs. 145). In responders to both clades, the overall magnitude of response to the clade B vaccine-matched panel was significantly higher (p<0.0001) than to clade C PTE panel (p<0.001); the same pattern held for gag (p = 0.006), pol (p<0.001), and nef (p<0.001) antigens [3].

The premature halt of Phambili, before the immunogenicity subset of participants reached third vaccination, potentially affected the detection of additional and/or stronger predictors. There may not be enough power to detect additional factors associated with the Phambili vaccine immune response or yield stronger associations linked to higher vaccine dosage.

While Step utilized the same vaccine regimen, it was not conducted in South Africa, and therefore differed from Phambili with respect to modes of HIV-1 transmission, risk, and clade; and background Ad5 seroprevalence [19]. Step has not yet been examined for potential predictors of immune response.

Step was conducted in a region exposed to circulating subtype B viral strains and included populations of high-risk homosexual men. Consistent with previous trials and Phambili, the MRK vaccine in Step was highly immunogenic for inducing HIV-specific T-cells. IFN-γ-secreting HIV-specific T-cells were detected ex-vivo by validated IFN-γ ELISpot and intracellular cytokine staining assays in 77.0% of Step vaccine recipients, with the majority recognizing two to three HIV proteins [20]. While Phambili was of smaller sample size, all vaccine recipients demonstrated immune response to ≥1 HIV antigen. In Step, those with lower baseline Ad5 titers (≤200 units) had higher immune response rates; overall immune responses did not differ between sexes [20]. Our study shows baseline NAd5 titer levels were not a statistically significant predictor of vaccine immune response, despite 80.6% of the vaccine recipients having detectable baseline Ad5 titer levels; response rates were higher in females. With dissimilar detectable Ad5 titer category cut-offs (200 units in Step vs. 18 in Phambili) there were possibly too few Ad5 seronegative Phambili participants to detect a relationship between baseline Ad5 titer and vaccine immune response. However, data from a comparative study of two MRK-Ad5/HIV-1 vaccine trials conducted in parallel (Step and HVTN 071) shows pre-existing adenovirus-specific CD4+ T-cell responses, in addition to NAd5 antibodies, dampen HIV-specific CD4+ and CD8+ T-cell responses induced by Ad5-vectored vaccination. Therefore, an Ad5 seronegative participant may still have high frequencies of pre-immunization adenovirus-specific CD4^+^ T-cells, which could impact the number of epitopes and magnitude of HIV-specific CD8^+^ T-cell responses [21].

There is supporting evidence to explain predictors of immune response found in our study; namely sex, BMI, and drinking.

There are sex differences in immune function. During reproductive years, women have stronger immune responses than men, thought to be controlled by differences in blood levels of gonadal steroid hormones [22]. Estrogen tends to stimulate immune responses, while testosterone is immunosuppressive [23]–[24]. Although estrogen is also present in males, the concentration is too low to affect immune responses [23].

Evidence explains a relationship between BMI and immune response. Data suggests levels of leptin, a cytokine-like hormone linked to the control of food intake and metabolism, is higher in obese individuals [25]. Leptin can act as a negative signal for proliferation of T-cells, suppressing the immune system and increasing incidence and severity of infectious disease [25].

Acute and chronic alcohol exposure exerts multiple effects on the immune system which may explain suppression of vaccine immune responses. Alcohol can impede early responses to infection, decrease production of most white blood cells, impair navigation ability of neutrophils to sites of injury and infection, and remove and/or alter macrophages and cytokines [23], [26]–[28]. People who drink excessively, also tend to neglect their own nutrition, which can cause the immune system to suffer [29].

In the context of other vaccine studies, the influence of these predictors has been noted. For example, one study which vaccinated older adults with Hepatitis A and B vaccines concluded that sex, BMI, and age significantly influenced both Hepatitis A and B vaccine immune responses [30]. However another study conducted with frail, elderly patients, failed to show an associate between BMI, age, and influenza vaccine antibody response [31]. Additionally, a third study reports that alcoholics have been shown to have poorer responses to some serotypes of the pneumococcal vaccine as compared to non-alcoholic controls [32].

There are possible interrelationships among sex, BMI, and alcohol consumption. Alcohol is related to BMI as it boosts cortisol levels, a fat-creating hormone; generally reducing fat metabolism by as much as 73% due to loss of muscle and dehydration [33]; and is correlated with irresponsible and over- eating [29]. Alcohol itself is high in calories with little nutritional value. All of these factors promote weight gain [29]. Both increased BMI and alcohol intake have been shown to affect circulating sex hormones - adipose (fat) tissue is a source of estrogen biosynthesis and chronic alcohol exposure can also alter production of estrogen and testosterone [23], [34].

Our study found a significantly suppressing association between overweight/obese BMI and clade C immune response, while the opposite was true for female sex. However, the majority of females enrolled had BMI above 24.99, defined as overweight, and most females had higher BMI than their male counterparts. The boosted vaccine-induced immune response might be due to estrogen playing an immunomodulatory role. Our Phambili females were all of reproductive age and adipose tissue further creates estrogen.

Identification of potential confounders is useful in understanding HIV vaccine immune responses and could help scientists develop more suitable vaccine candidates for target populations [35]. The aforementioned explanations for the observations of the predictors are our speculations, and further investigations are needed to ascertain how predictors play a role in modifying each other. A limitation of this study is that it is not possible to gauge the effect of all possible confounders. Vaccination dosing and inoculation spacing should be explored in relation to obesity as a predictor of vaccine immune response. While our data suggests baseline NAd5 antibodies do not affect immune response, pre-existing immunity to the vaccine vector might still dampen other predictors' HIV-insert specific responses and/or alter efficacy [20]–[21].

Our study suggests overweight/obese BMI suppresses immune responses to the Phambili vaccine regimen, as obesity generally suppresses the immune system [14], [36], and creates an emerging concern in the field of vaccine efficacy. A recent meta-analysis shows a trend toward greater obesity in Southern Africa in the past three decades [37]. In 2008, 50.0% of men and 60.0% of women were overweight in South Africa, pointing towards an obesity pandemic [37]. Stratification by sex and BMI should be considered in future vaccine efficacy trials to assess the effects on HIV-1 vaccines by these predictors.
